# Spontaneous Resolution of Mirror Syndrome following Demise of Hydropic Twin

**DOI:** 10.1155/2012/783408

**Published:** 2012-10-11

**Authors:** Kate E. Oliver, Kimberly W. Hickey, Scott M. Petersen

**Affiliations:** Prenatal Assessment Center, Department of Obstetrics and Gynecology, Walter Reed National Military Medical Center, 8901 Wisconsin Avenue, Bethesda, MD 20889, USA

## Abstract

Maternal mirror syndrome is a rare consequence of fetal hydrops. By convention, delivery is recommended in pregnancies complicated by mirror syndrome due to grave fetal prognosis. We describe a case of a dichorionic, diamniotic twin gestation complicated by hydrops fetalis of twin B. The patient declined selective feticide. Two weeks later, intrauterine fetal demise of fetus B was diagnosed and complete resolution of mirror syndrome followed. Unaddressed, mirror syndrome can lead to significant maternal and fetal complications. This case illustrates resolution of mirror syndrome following spontaneous intrauterine demise of the hydropic fetus.

## 1. Introduction

Mirror syndrome is a rare consequence of severe fetal hydrops of both immune and nonimmune etiologies, with fewer than 100 cases previously described in the literature [1]. JW Ballantyne reported the first case in 1892, and subsequently, cases have been published using a variety of terms, including Ballantyne's syndrome, maternal hydrops syndrome, pseudotoxemia, and triple edema syndrome, among others [2–7]. The etiology of mirror syndrome remains unclear, although an underlying severe fetal hydrops is proximate to the development of the condition [2, 5]. Mirror syndrome traditionally was diagnosed in cases where fetal hydrops evolved in the setting of an underlying fetal anemia, typically secondary to rhesus isoimmunization [2, 4–6]. More recently, cases of mirror syndrome have been reported as a result of fetal hydrops stemming from nonimmune etiologies, such as fetal structural anomalies [6], cardiac abnormalities [2], fetal arrhythmias [4], and intrauterine infection [3].

The development of mirror syndrome portends poor prognosis and impending fetal death [2, 3, 6]. Uncommonly, mirror syndrome has resolved with treatment and improvement of the hydrops of the affected fetus, although some causes are inherently uncorrectable [3, 5, 6]. In one recent account, maternal mirror syndrome arising from a parvovirus B19 infection resolved with spontaneous resolution of the associated fetal hydrops [3]. Another case report demonstrated resolution of mirror syndrome with selective reduction of the affected twin [8], while a third case of mirror syndrome resolved with correction of the fetal tachyarrhythmia [4]. To our knowledge, there are no reported cases of resolution of mirror syndrome with spontaneous demise of the affected fetus. Should maternal mirror syndrome fail to resolve either spontaneously, with selective fetal reduction, or following treatment aimed at correcting the underlying fetal etiology of the hydrops, delivery is usually obligatory for maternal indications [6, 8].

## 2. Case

We present a case of a 38-year-old gravida 4 para 1021 with dichorionic-diamniotic IVF/ICSI twin gestation. The patient's past medical and surgical histories were unremarkable. Her obstetrical history was notable for a prior IVF gestation which resulted in a spontaneous vaginal delivery at term. At her initial prenatal visit at 9-week estimated gestational age, the patient's blood pressure was noted to be 122/65 mmHg, and her most recent prepregnancy weight was 172 pounds. Her blood type was O positive with a negative antibody screen. Her initial hemoglobin was 12.4 g/dL with a hematocrit of 35.9%. Her antenatal laboratory values were otherwise normal. The patient declined maternal serum screening and amniocentesis for advanced maternal age. At 17-week estimated gestational age, a level II ultrasound revealed normal anatomy for both fetuses, confirming the chorionicity to be dichorionic-diamniotic. No markers for aneuploidy were identified in either fetus.

At 23-week estimated gestational age, repeat growth scan revealed adequate growth of twin A with severe hydrops of twin B. The ultrasonographic findings included a 50% intertwin discordance in estimated weight, bilateral severe pleural effusions, severe pericardial effusion, severe ascites, and a normal-appearing heart. The differential diagnosis for fetal hydrops in this patient included intrauterine infection, congenital heart disease or other anatomic anomaly, or another unknown cause. Isoimmunization was unlikely in the setting of a normal antibody screen, and no ABO incompatibility existed as both parents were O blood type. Investigation into the etiology of the hydrops was initiated, including serum testing, amniocentesis, and fetal echocardiography. The patient underwent maternal serum testing to include TORCH titers and hemoglobin electrophoresis, and diagnostic amniocentesis for karyotype, FISH, and PCR for HSV, CMV, parvovirus B12, and toxoplasmosis. Diagnostic amniocentesis and maternal serum testing excluded infectious and chromosomal etiologies, while fetal echocardiography revealed an atrioventricular canal malformation as the likely etiology of the hydrops. The patient was counseled regarding the 70% risk of fetal demise of twin B and the associated risks to twin A, and she declined selective fetal reduction.

The patient presented at 24-week estimated gestational age with worsening abdominal distention, dyspnea, and abdominal pressure and underwent therapeutic amniocentesis for worsening polyhydramnios. A betamethasone course was completed due to concern for early delivery. Repeat level II ultrasound four days later revealed normal amniotic fluid volume following amnioreduction and was remarkable only for hydrops, with no anomalies visualized. Within one week, at 24-week 6-day estimated gestational age, the patient presented to her scheduled prenatal visit with worsening edema, abdominal distention, and shortness of breath. The patient had gained 10 pounds since her last obstetrics visit approximately 4 weeks prior to presentation, and clinic blood pressure was noted to be 140/82 mmHg. The worsening dyspnea, increasing edema, and polyhydramnios in the setting of a mild range blood pressure raised concern for mirror syndrome. Initial laboratory values revealed a 24-hour urine protein of 360.4 mg/24 hr with normal aminotransferases, a serum creatinine of 0.6 mg/dL, uric acid of 5.3 mg/dL, a slightly elevated LDH of 250 U/L, and a hemoglobin of 10.4 g/dL with a hematocrit of 30.1%. Two weeks after initial diagnosis of severe fetal hydrops, intrauterine fetal demise of fetus B was diagnosed. A 24-hour urine protein collection performed that day revealed a total urine protein of 680.4 mg/24 hr. Serum LDH and uric acid values were elevated at 254 U/L and 6.3 mg/dL, respectively.

The patient was admitted to the antepartum service for inpatient bedrest and close monitoring. While on bedrest, the patient remained normotensive and serial laboratory values demonstrated an improvement in the abnormalities of LDH and uric acid. Fetal testing of twin A remained reassuring. Given the improved clinical picture, the patient was discharged to home on bedrest with close followup. One week after the diagnosis of the fetal demise of twin B, a repeat 24-hour urine protein excretion revealed significant improvement, with the total urine protein 313 mg/24 hr, and the patient endorsed symptomatic improvement. Within two weeks of the demise, the patient's mirror syndrome had resolved completely. Repeat 24-hour urine protein collection revealed a total protein excretion of 275.5 mg/24 hr. Serum LDH and uric acid values were trending toward normal, and the patient's coagulation studies revealed no evidence of disseminated intravascular coagulation. The patient's massive polyhydramnios and edema improved, with a 7-pound weight loss over the course of two weeks. The patient has currently surpassed 29-week estimated gestational age with reassuring fetal testing and adequate fetal growth of the remaining twin.

## 3. Discussion

Mirror syndrome, a rare but serious consequence of pregnancies affected by fetal hydrops of any cause, has been described infrequently in the literature, and the incidence of the disease is unknown [3, 6]. The syndrome classically includes massive anasarca, proteinuria, and mild hypertension in the setting of fetal hydrops [2, 3, 6]. Other features include hemodilution, which distinguishes mirror syndrome from preeclampsia, and elevated serum uric acid [2, 6]. Preeclampsia is associated with the syndrome, though only present in about half of cases, and eclampsia is rare [2, 7].

The pathogenesis of mirror syndrome has not been elucidated, although a placental origin seems likely [3, 5, 7]. Placental ischemia is presumed to be central to the development of preeclampsia, and in mirror syndrome, it may develop secondary to the edema characteristic of the disease [5, 7, 8]. Placental ischemia is thought to initiate the cascade mediated by antiangiogenic factors which may lead to the sequelae of both disorders [5, 7]. Further research into elevations of serum markers of placental dysfunction is needed to better elucidate the underlying etiology of mirror syndrome [5, 7].

Untreated, mirror syndrome leads to significant maternal and fetal morbidity and mortality, and traditionally, maternal disease is addressed by delivery [3, 6, 8]. This case illustrates complete resolution of maternal mirror syndrome with a noncorrectable lesion following intrauterine demise of the hydropic fetus. Despite counseling regarding the likely lethal severity of the cardiac anomaly underlying twin B's hydrops, our patient declined selective reduction; conservative management was thereby compulsory. Resolution of the maternal manifestations of the syndrome occurred within two weeks of the diagnosis of the intrauterine demise of the hydropic twin. This clinical improvement, in keeping with the presumed role of the placenta in the progression of the disease, correlates well with the timing of placental involution that has been previously documented [3, 8]. The resolution of mirror syndrome in this case allowed for continuation of the pregnancy to the benefit of the unaffected twin, allowing for fetal maturation to the current 29-week estimated gestational age in a pregnancy which may have otherwise been delivered for maternal indications.
